# Hidden players in plant response to sulfur deficit and beyond: insights into the function of the proteins Sulfur Deficiency Induced and Response to Low Sulfur

**DOI:** 10.1093/jxb/erag083

**Published:** 2026-02-16

**Authors:** Agnieszka Sirko, Anna Wawrzyńska, Marcin Olszak, Justyna Piotrowska, Marzena Sieńko

**Affiliations:** Institute of Biochemistry and Biophysics, Polish Academy of Sciences, Warsaw 02-106, Poland; Institute of Biochemistry and Biophysics, Polish Academy of Sciences, Warsaw 02-106, Poland; Institute of Biochemistry and Biophysics, Polish Academy of Sciences, Warsaw 02-106, Poland; Institute of Biochemistry and Biophysics, Polish Academy of Sciences, Warsaw 02-106, Poland; Institute of Biochemistry and Biophysics, Polish Academy of Sciences, Warsaw 02-106, Poland; Michigan State University, USA

**Keywords:** Arabidopsis, crop improvement, glucosinolates, *LSU* gene family, nutrient signaling, redox regulation, *SDI* gene family, sulfur deficiency, sulfur metabolism, sulfur use efficiency

## Abstract

Sulfur is an essential macronutrient for plants, playing a central role in diverse metabolic processes. Under sulfur-deficient conditions, plants undergo extensive transcriptional reprogramming, which includes the induction of numerous genes encoding proteins of largely unknown function. Among these, two protein families have been studied more intensively: Sulfur Deficiency Induced (SDI) and Response to Low Sulfur (LSU). This review summarizes current experimental evidence on their involvement in sulfur assimilation, stress responses, and overall plant adaptation to sulfur scarcity. Special emphasis is placed on the LSU family, where recent evidence suggests that the function of these proteins extends beyond sulfur starvation responses, pointing to broader roles in plant growth, development, and resilience to diverse stresses.

## Teaser

This review focuses on two families of genes described as sulfur deficiency markers, *SDI* and *LSU*, and describes different strategies plant utilize to cope with sulfur shortage.

## Introduction

In nature, sulfur (S) may be found in both organic and inorganic forms. Microbes and plants convert inorganic sulfate into sulfide, which they subsequently incorporate into organic compounds. Animals rely on dietary sources of S because they cannot reduce sulfate to sulfide and incorporate it into organic S compounds, which makes plants the primary source of organic S for animals (Leustek *et al.*, 2000; Droux, 2004). Consequently, the plant–environment S cycle has a major impact on human health and nutrition (Nakai and Maruyama-Nakashita, 2020). The S cycle is completed when microbes decompose organic S present in plant and animal residues, converting it back into inorganic sulfate (Takahashi *et al.*, 2011) (Fig. 1).

**Fig. 1.**
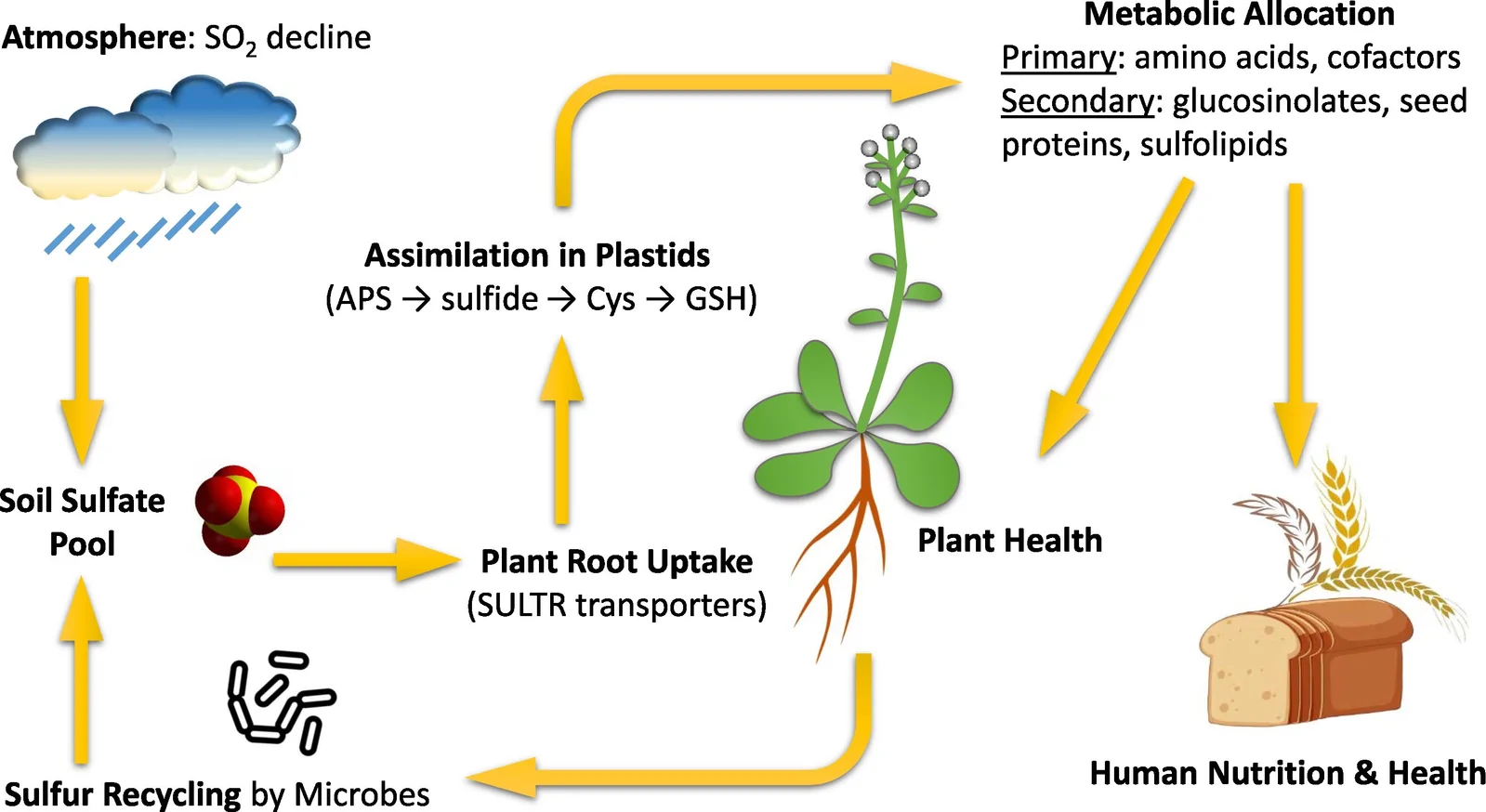
Overview of sulfur cycling and its metabolism in plants. Abbreviations: APS, adenosine 5′-phosphosulfate; Cys, cysteine; GSH, glutathione; SULTR, sulfate transporter.

S is required for the synthesis of numerous vital biomolecules, including amino acids, cofactors, and secondary metabolites. Both biotic and abiotic stress responses require compounds that contain S. Glutathione (GSH), for instance, is essential for reactive oxygen species (ROS) detoxification and acts as a substrate for the production of phytochelatins, compounds crucial for heavy metal detoxification (Cobbett, 2000; Pastore *et al.*, 2003). Furthermore, the *Brassicaceae* family's S-rich secondary metabolites, glucosinolates (GSLs) as well as camalexin, serve as defensive mechanisms against pathogens and herbivores (Jacoby *et al.*, 2021). These compounds also give this plant family its distinctive smells and scents, and they have several health benefits for people (Talalay and Fahey, 2001). An example from another plant family, the *Amaryllidaceae*, is allicin, produced by garlic, which exhibits a wide range of pharmacological properties, including neuroprotective, anti-Alzheimer’s, anti-inflammatory, anticancer, antioxidant, cardioprotective, and antidiabetic properties (Deng *et al.*, 2023). S is also necessary for the production of plant hormones, including ethylene and abscisic acid (ABA), which regulate plant growth and development and are involved in seed germination, drought tolerance, and plant senescence (Hasanuzzaman *et al.*, 2018).

The sulfate transport system, the assimilation pathway, and the subsequent metabolism of S-containing compounds are highly complex processes that have been the focus of numerous comprehensive reviews, both earlier and more recent (Lewandowska and Sirko, 2008; Aarabi *et al.*, 2020; Watanabe *et al.*, 2021). Briefly, these processes encompass the uptake of sulfate from the environment, its activation and reduction to sulfide, and the incorporation of reduced S into amino acids (cysteine and methionine) and other essential S metabolites. Given the breadth of these topics and the extensive literature available, they will not be discussed in detail in this review.

## Nutritional and adaptive implications of S deficiency in plants

S plays a crucial role in determining both crop yield and quality. The content of S-containing amino acids in plants is an important indicator of crop nutritional value. S deficiency decreases the levels of these essential amino acids in grains, whereas S supplementation promotes the synthesis of S-containing proteins, thereby enhancing crop quality and nutritional content (Yu *et al.*, 2018; Li *et al.*, 2020). In recent decades, the availability of S in the environment has declined, largely due to reduced atmospheric emissions of sulfur dioxide (SO₂) from industry, the widespread use of S-free fertilizers, and the lowering of S content in fuels (Feinberg *et al.*, 2021). Consequently, S deficiency has become a major limiting factor in agriculture production.

S deficiency exerts a dual impact on plant biology and agriculture: it lowers the nutritional quality of crops while simultaneously triggering adaptive responses at both molecular and physiological levels. On one hand, insufficient S restricts the accumulation of S-containing amino acids and proteins, negatively affecting yield and food quality. It is well documented that wheat seed storage proteins—such as gliadins and glutenins, which confer the characteristic rheological properties of dough—play a central role in determining wheat processing quality (Peng *et al.*, 2022). S enhances the accumulation of free glycine at the onset of wheat grain filling and subsequently promotes its incorporation into glutenin (Yu *et al.*, 2021). Among crops of major agro-economic importance, oilseed rape (used for oil production, animal feed, and as a potential biofuel source) is particularly noteworthy. Like most members of the *Brassicaceae* family, it has higher S requirements than other crop species and is therefore especially sensitive to S deficiency, which can reduce yield by up to ∼40% (Abdallah *et al.*, 2010). On the other hand, plants respond to S deficiency by activating complex regulatory networks that integrate environmental cues with metabolism, growth, and development, thereby optimizing S uptake and utilization (Nakai and Maruyama-Nakashita, 2020; Ristova and Kopriva, 2022; Canales *et al.*, 2023).

## Molecular and physiological regulation of plant responses to S deficit

### Phenotypic level

S deficiency in plants leads to characteristic phenotypic changes that have been well documented across species. Typical symptoms include interveinal chlorosis, reduced growth rates, and delayed or impaired reproductive development (Hawkesford and De Kok, 2006). These visible traits reflect the systemic impact of S limitation on primary metabolism and protein synthesis, particularly due to the depletion of S-containing amino acids such as cysteine and methionine.

Changes in root morphology under S deficiency have also been extensively investigated (Kutz *et al.*, 2002; Dan *et al.*, 2007; Gruber *et al.*, 2013; Dong *et al.*, 2019; Joshi *et al.*, 2019). In Arabidopsis, S deprivation induces an increase in root hair number, modifications in root branching, and altered lateral root development, all mediated by hormonal and metabolic pathways (Kutz *et al.*, 2002). These adjustments enhance sulfate acquisition under nutrient stress. A notable increase in the root-to-shoot ratio is also a typical response to S shortage, as shoot growth is inhibited more strongly than root growth—a pattern more pronounced than that observed under other nutrient deficiencies (Gruber *et al.*, 2013).

### Proteomics and metabolomics

Compared with transcriptomic studies, proteomic and metabolomic investigations of S deficiency remain relatively limited, yet they provide crucial complementary insights. Multi-omics approaches (transcriptomics+proteomics+metabolomics) are essential to capture the dynamic regulation of S metabolism. S-responsive transcript abundance may not directly translate into metabolic outcomes or protein levels (Nikiforova *et al.*, 2005). Sulfate transporter proteins (SULTR1;1, SULTR1;2) accumulate during S limitation even when their transcript levels are not tightly correlated across all time points of S starvation, highlighting post-transcriptional control of protein levels (Yoshimoto *et al.*, 2007). The combined proteomic and transcriptomic results indicate that seeds regulate storage protein composition through multiple layers, including mRNA expression changes and protein processing modifications, to adapt to S stress (Higashi *et al.*, 2006). Early integrative studies combining transcriptomics and metabolomics demonstrated that sulfate deprivation induces the accumulation of *O*-acetylserine (OAS), which functions both as a metabolic intermediate and as a signaling molecule that reports S status to the transcriptional machinery (Hirai *et al.*, 2003; Nikiforova *et al.*, 2003). High-throughput metabolomic studies have explored the metabolic changes associated with S deficiency in whole seedlings (Zhang *et al.*, 2011; Bielecka *et al.*, 2015) and in separated shoot and root (Hirai *et al.*, 2003; Hubberten *et al.*, 2012; Forieri *et al.*, 2017). Typical metabolic responses include a pronounced reduction in S metabolites, such as GSLs, and a moderate decrease in GSH, cysteine, methionine, and *S*-adenosylmethionine. In contrast, non-polar amino acid levels generally increase (Forieri *et al.*, 2017). S deficiency also leads to decreased glucose, sucrose, and maltose contents in shoots. Low glucose and S limitation—perceived via sulfide or sulfite sensing—are relayed to the Target of Rapamycin (TOR) kinase pathway, which subsequently reduces TOR activity, leading to suppressed translation and growth retardation (Dong *et al.*, 2022).

More recent proteomic analyses have revealed changes in redox-associated proteins and central metabolic enzymes under S limitation, highlighting the importance of post-transcriptional regulation (Liu *et al.*, 2014). Despite these advances, large-scale proteome datasets remain limiting, representing a key direction for future studies (Watanabe and Hoefgen, 2019).

### Approaches used to identify S-deficiency-regulated genes

A variety of molecular and genomic approaches have been employed to identify genes regulated by S deficiency in plants, particularly in Arabidopsis. Among these, transcriptome-wide analyses have been the most informative, enabling global profiling of gene expression changes in response to S deprivation.

Pioneering macroarray/microarray studies at the beginning of this century established the foundation for understanding transcriptional responses to S limitation (Hirai *et al.*, 2003; Maruyama-Nakashita *et al.*, 2003; Nikiforova *et al.*, 2003). Although transcriptional changes vary depending on the duration and severity of S deprivation, certain S deficiency marker genes are consistently induced, including genes encoding sulfate transporters (*SULTR1;1*, *SULTR1;2*), adenosine 5′-phosphosulfate (APS) reductase (*APR*) isoforms, and several genes of unknown function.

Subsequent microarray and RNA-seq studies have expanded this understanding in Arabidopsis (Hirai *et al.*, 2005; Maruyama-Nakashita *et al.*, 2005; Iyer-Pascuzzi *et al.*, 2011; Bielecka *et al.*, 2015; Aarabi *et al.*, 2016; Forieri *et al.*, 2017) and crop species such as tobacco and tomato (Lewandowska *et al.*, 2010; Canales *et al.*, 2020), revealing tissue-specific and time-dependent transcriptional responses. These investigations not only identified new S-responsive genes but also uncovered regulatory mechanisms, including novel *cis*-regulatory motifs such as the SURE element and UPE-box, which mediate S-dependent transcription (Maruyama-Nakashita *et al.*, 2005; Wawrzynska *et al.*, 2010). Transcriptome analyses of mutants have been instrumental in validating the role of specific regulators. For example, studies on mutants lacking the master regulator Sulfur Limitation 1 (SLIM1) demonstrated that transcription factors from the EIN3-like family play a central, but not exclusive, role in coordinating S deficiency responses (Maruyama-Nakashita *et al.*, 2006; Dietzen *et al.*, 2020).

Beyond individual datasets, meta-analyses have extracted conserved signatures of S deficiency. Integration of multiple Arabidopsis transcriptomes revealed that S-deficiency-regulated genes are enriched not only in S metabolism but also in pathways linked to carbon and nitrogen metabolism, cell wall organization, and proteolysis (Henríquez-Valencia *et al.*, 2018). Such integrative analyses, supported by advanced bioinformatic tools, have helped identify transcription factor networks and novel candidate regulators not apparent in single studies. Comparative RNA-seq analyses across Arabidopsis, tomato, and rice further enabled the construction of gene regulatory networks and elucidated cascades controlling sulfate transporters and metabolic genes (Fernández *et al.*, 2024).

While transcriptomics has been indispensable, it alone cannot fully explain the complexity of plant responses to S limitation. The integration of multi-omics approaches, including transcriptomics, proteomics, and metabolomics, has provided deeper insights into how plants sense and adapt to sulfate scarcity (Watanabe and Hoefgen, 2019). Comparative analyses combining transcriptome and metabolome data have also identified transcription factors responsive to both S deprivation and resupply, revealing novel links between S, nitrogen, and phosphorus nutritional signaling (Bielecka *et al.*, 2015).

Although this growing body of data has substantially advanced our understanding of gene regulation under S deficiency, relatively few regulatory mechanisms have been fully characterized. Identifying the sensors and signaling components that orchestrate S deficiency responses remains a critical challenge. Uncovering these elements will not only refine our molecular understanding but may also reveal novel targets for improving crop resilience and nutritional quality.

Transcriptome-wide studies have revealed that S deficiency affects the expression of a diverse set of genes. The encoded proteins can be broadly classified into groups related to sulfate transport, hormonal regulation, secondary metabolism, transcriptional control, and unknown function (Table 1). Notably, the biological functions of approximately 45% of the differentially expressed genes under S-deficient conditions remain unknown (Henríquez-Valencia *et al.*, 2018).

**Table 1.** Classification of representative S-deficiency-induced genes

Category	Examples	Role	References
S transport	*SULTR1;1*, *SULTR1;2*, *SULTR4;1 SULTR4;2*	Uptake and transport systems	Maruyama-Nakashita *et al.* (2003); Kataoka *et al.* (2004)
Transcription factors	*MYB28*	Activation of glucosinolate biosynthesis	Hirai *et al.* (2007); Bielecka *et al.* (2015)
	*RVE2*	Circadian-regulated transcription factor involved in S-deficiency response	Bielecka *et al.* (2015); Henríquez-Valencia *et al.* (2018)
Secondary metabolism	*BGLU28*	β-Glucosidase involved in glucosinolate turnover	Sugiyama *et al.* (2021)
Hormonal pathways	*LOX2*, *OPR*	Enzymes in jasmonic acid biosynthesis	Maruyama-Nakashita *et al.* (2003); Nikiforova *et al.* (2003)
	*NIT3*	Nitrilase involved in auxin biosynthesis	Kutz *et al.* (2002); Nikiforova *et al.* (2003)
Unknown	At3g05400	Major facilitator superfamily protein	Henríquez-Valencia *et al.* (2018)
	At4g31330	Transmembrane protein	Henríquez-Valencia *et al.* (2018)
	At5g40670	Transmembrane protein	Henríquez-Valencia *et al.* (2018)
	At2g34655	Hypothetical protein	Dietzen *et al.* (2020)
	At2g32487	Hypothetical protein	Dietzen *et al.* (2020)

### Why focus on proteins of unknown function?

Despite extensive research on canonical S metabolism, a significant proportion of S-deficiency-regulated genes encode proteins with unknown function. These genes often display the strongest induction upon S withdrawal, suggesting essential but non-canonical roles in the adaptive response. Some of these proteins could be novel enzymes, transporters, or regulators specifically adapted for S acquisition or utilization. Recent studies exemplify how functional characterization of such genes can change our understanding of S metabolism regulation. In this review we will focus on two different families of such proteins, Sulfur Deficiency Induced (SDI) and Response to Low Sulfur (LSU). SDI proteins were initially unannotated stress markers, but later proved to be repressors of GSL and seed storage protein biosynthesis (Aarabi *et al.*, 2016, 2021). Similarly, LSU proteins—once considered enigmatic—are now recognized as molecular hubs coordinating redox regulation, hormone signaling, and selective autophagy (Canales *et al.*, 2023; Niemiro *et al.*, 2024; Piotrowska *et al.*, 2024b). These examples underscore the importance of investigating uncharacterized proteins. They often reveal previously unknown regulatory layers of nutrient signaling and may offer novel biotechnological tools for engineering crops with enhanced S use efficiency or stress tolerance.

## The SDI family

The name of the *SDI* gene family derives from its specific induction under S deficiency. *SDI1* was first identified as an indicator of S status in *Triticum aestivum* field studies of multiple nutrient deficiencies (Howarth *et al.*, 2009). Subsequently, five putative *SDI* genes were identified in Arabidopsis through nucleotide sequence homology searches. Microarray analyses confirmed that, as in wheat, the gene with the highest sequence similarity to *SDI1* (At5g48850) was strongly induced by S deprivation (Maruyama-Nakashita *et al.*, 2005). Detailed studies of *SDI1* expression revealed that it is among the most rapidly induced genes responsive to S deficiency (Howarth *et al.*, 2009). Investigations into natural diurnal oscillations (Espinoza *et al.*, 2010) and responses to light and temperature (Caldana *et al.*, 2011) showed specific periods of accumulation of OAS—a key metabolite that signals S deficiency. *SDI1* and *SDI2* were found to belong to a group of six genes, termed the OAS cluster, that are co-expressed with rising OAS levels (Hubberten *et al.*, 2012).

Recent analysis indicates that there are six members of the *SDI* family in Arabidopsis: AT5G48850 (*SDI1*), AT1G04770 (*SDI2*), AT4g20900 (*Male Sterile 5* or *Three Division Mutant1* (*MS5/TDM1*)), AT5G44330 (*MS5-like*), AT3G51280, and AT5G22794 (Aarabi *et al.*, 2020). All of the encoded proteins except AT5G22794 contain characteristic tetratricopeptide repeat (TPR) domains, which mediate protein–protein interaction (Lamb *et al.*, 1995). While the functions of MS5, SDI1, and SDI2 are known, the functions of the remaining three proteins remain largely uncharacterized.

### Functional diversity and regulatory roles of SDI family proteins in S metabolism

The first characterized protein within the SDI family was MS5. It plays a crucial role in regulating translation within pollen. During the second meiotic division, MS5 is incorporated into processing bodies through interaction with Suppressor With Morphogenetic Effects On Genitalia 7 (SMG7). Subsequently, MS5 recruits eIF4F, the primary translation initiation complex, transiently trapping it within processing bodies and thereby suppressing translation (Bulankova *et al.*, 2010; Cairo *et al.*, 2022). Mutants deficient in MS5 fail to terminate male meiosis and do not form microspores, and hence the male sterility of *ms5* (Glover *et al.*, 1998). The role of its closest homolog, MS5-like, is as yet unknown.

Systemic changes during S deficiency involve metabolic reprogramming that deprioritizes S-consuming processes, such as GSL biosynthesis in *Brassicaceae* and storage protein accumulation in seeds. Central to these adjustments are two SDI proteins, SDI1 and SDI2, whose expression is specifically up-regulated under S-deficient conditions (Aarabi *et al.*, 2016). The first major insight into the functional relevance of SDI proteins came from the discovery that SDI1 binds to and inhibits activation capacity of the transcription factor Myeloblastosis 28 (MYB28), a master positive regulator of aliphatic GSL biosynthesis (Aarabi *et al.*, 2016). GSLs are defense compounds rich in S, and their synthesis represents a substantial sink for this nutrient. However, electrophoretic mobility shift assays showed that the SDI1–MYB28 interaction does not affect the DNA-binding ability of MYB28, suggesting that SDI1 disrupts interactions with other transcription factors or with RNA polymerase (Aarabi *et al.*, 2016). In Arabidopsis, SDI proteins redirect S allocation from secondary metabolism toward essential primary metabolites. This repression mechanism represents an elegant regulatory node integrating environmental nutrient status with metabolic priorities and highlights the versatility of SDI proteins as repressors capable of rapidly adjusting metabolic programs in response to external cues.

Beyond vegetative tissues, SDI proteins also play active roles during seed development. Aarabi *et al.* (2021) demonstrated that under S limitation, SDI1 modulates the composition of seed storage proteins by reducing S-rich 2S albumins and increasing S-poor 12S globulins. This shift in protein allocation likely maintains seed viability while preserving internal S reserves. Notably, these alterations occur without a significant reduction in total protein content, indicating a highly selective and finely tuned reprogramming of gene expression. Interestingly, to exert its inhibitory effect, SDI1 forms a ternary complex with MYB28 and MYC2, the latter being a positive regulator of seed storage protein accumulation (Gao *et al.*, 2016). Such molecular plasticity highlights the functional versatility of SDI proteins as modulators that integrate developmental and environmental signals.

Recent studies suggest that SDI1 not only suppresses the accumulation of GSLs and 2S seed storage proteins under S-deficient conditions, but also specifically reduces the levels of the sulfolipid sulfoquinovosyldiacylglycerol (SQDG), while leaving other chloroplast membrane lipids unaffected (Aarabi *et al.*, 2023). Although the underlying mechanism remains unclear, evidence indicates that SDI1 negatively regulates the expression of *UDP-Glucose Pyrophosphorylase 3* (*UGP3*) gene, which encodes the first enzyme in the SQDG biosynthetic pathway. Collectively, these findings highlight the specific role of SDI1 under S deficiency, revealing its capacity to coordinately down-regulate the three major S pools in plants—GSLs, 2S-rich seed storage proteins, and SQDGs. While SDI1 appears to be the predominant functional protein, SDI2 may act redundantly or exhibit tissue-specific activity during S deficiency. Furthermore, a meta-analysis of *cis*-regulatory elements in the promoters of SDI1 and SDI2 suggests that these genes may also function beyond S deficiency conditions and likely fulfill distinct, non-overlapping roles (Rakpenthai *et al.*, 2022).

Although the functions of other members of this protein family remain largely unknown, AT3G51280 has been shown to be expressed in root zone 1 at the root tip, where genes related to cell division, DNA metabolism, and the cell cycle show elevated expression, suggesting a potential role in these processes (Krishnamurthy *et al.*, 2018).

### Evolutionary perspectives and functional redundancy

S requirements vary among plant species, with *Brassicaceae* identified as the most S-dependent among crop families. This variation suggests that individual plant species have evolved distinct mechanisms to maintain S homeostasis under S-deficient conditions. Moreover, the presence of SDI homologs in both monocots and non-*Brassicaceae* dicots indicates that SDI proteins may perform species-specific functions—an area that warrants further investigation. Several independent studies have confirmed S-deficiency-induced expression of *SDI* genes in tomato (*Solanum lycopersicum*) (Canales *et al.*, 2020), pea (*Pisum sativum*) (Henriet *et al.*, 2021), rice (*Oryza sativa*) (Wang *et al.*, 2022), and einkorn wheat (*Triticum monococcum*) (Bonnot *et al.*, 2020). The promoters of genes within this clade more frequently contain a *cis*-acting element known as the UPE-box (Wawrzynska *et al.*, 2010). Members of the EIL transcription factor family, including SLIM1—a master regulator of the S deficiency response (Maruyama-Nakashita *et al.*, 2006; Dietzen *et al.*, 2020), have been shown to bind to this element (Wawrzynska *et al.*, 2010; O’Malley *et al.*, 2016).

The strategic suppression of non-essential S sinks by SDI proteins presents promising opportunities for crop improvement, particularly in S-deficient soils. Engineering crops to modulate SDI activity could enhance S-use efficiency, improve seed nutritional quality, or enable targeted modification of secondary metabolite production. However, caution is warranted, as excessive SDI expression may compromise defense or developmental processes.

## The LSU family

One of the hallmark responses to S starvation in Arabidopsis is the induction of a group of genes collectively known as the *Response to Low Sulfur* (*LSU*) gene family (Sirko *et al.*, 2015; Canales *et al.*, 2023). Initially annotated as genes of unknown function, *LSU* genes have since emerged as important players in S homeostasis and stress signaling pathways. The LSU family has drawn increasing attention due to its unique expression profile under S-limited conditions and its potential regulatory roles extending beyond S metabolism. Recent studies, including work on the *lsu* quadruple mutant, have revealed that LSU proteins participate in extensive protein–protein interaction networks and contribute to stress acclimation and the integration of metabolic signals (Piotrowska *et al.*, 2024a, b).

### Discovery and characterization of *LSU* genes in Arabidopsis and other species

The *LSU* gene family encode a group of small proteins and were originally identified in Arabidopsis as being strongly up-regulated under S starvation (Sirko *et al.*, 2015). These genes, initially annotated as encoding proteins of unknown function, gained attention because of their dramatic transcriptional induction under S-limited conditions. The Arabidopsis genome contains four *LSU* genes, designated *LSU1–LSU4* (*At3g49580*, *At5g24660*, *At3g49570*, and *At5g24655*, respectively), each sharing a conserved gene structure (Sirko *et al.*, 2015). *LSU1*, *LSU2*, and *LSU3* also display similar expression patterns. Parallel research in *Nicotiana tabacum* identified a homologous gene family known as *Upregulated by Sulfur Deficit 9* (*UP9*), consisting of at least six members (*UP9A–UP9F*), among which *UP9C* was the most responsive to S deficiency (Lewandowska *et al.*, 2010). The first evidence for physiological significance of LSU-like proteins in tobacco came from analysis of a transgenic lines with reduced *UP9* expression, which revealed broad transcriptomic deregulation in response to S deficiency. Notably, more than 50% of S-responsive genes failed to be appropriately induced in these lines, suggesting a central role for UP9C in S-dependent signaling networks (Lewandowska *et al.*, 2010).

Beyond Arabidopsis and tobacco, *LSU-like* genes have been identified across a wide range of seed plants. A comprehensive phylogenomic analysis revealed 270 LSU homologs across 134 seed plant species, encompassing both monocotyledonous and dicotyledonous taxa (Uribe *et al.*, 2022). These homologs cluster into three major clades: two specific to dicots and one to monocots. Interestingly, *LSU* genes appear to be absent in non-seed-plant lineages such as bryophytes or algae, suggesting that LSU family members are a relatively recent evolutionary innovation within vascular plants. Gene copy number varies significantly among species, ranging from one to nine *LSU-like* genes per genome. In monocots such as wheat (*Triticum aestivum*), rice (*Oryza sativa*), and *Brachypodium distachyon*, *LSU* homologs are transcriptionally regulated in response to S availability, similarly to Arabidopsis *LSU* genes (Tombuloglu *et al.*, 2016; Uribe *et al.*, 2022). Sequence alignment analyses have revealed five conserved motifs within LSU proteins, supporting the existence of a structurally and functionally coherent protein family (Uribe *et al.*, 2022). These conserved features, together with the presence of S-responsive *cis*-elements (e.g. SURE-box) in promoter regions, point to a shared regulatory and functional framework among LSU homologs across plant species.

A major breakthrough in understanding LSU function in Arabidopsis came from the generation and characterization of the *lsu* quadruple mutant (Piotrowska *et al.*, 2024a). Plants lacking all four *LSU* genes, although they do not show a visible phenotype, exhibited defects in S uptake and metabolism, strongly implicating LSU proteins in maintaining S homeostasis. These mutants also displayed altered expression of key S assimilation genes, including *SULTR1;2* and *APR3*, indicating that LSU proteins participate in the regulation of the S assimilation pathway at both transcriptional and physiological levels.

Together, these discoveries establish the LSU family—and its tobacco counterparts UP9—as a novel class of S-responsive regulators, whose functions are closely linked to nutrient homeostasis, despite their initially enigmatic molecular identity.

### Expression patterns under S-deficient conditions

In Arabidopsis, the expression of *LSU1* and *LSU2* is strongly up-regulated during S starvation, with transcript levels increasing within 24 h of sulfate withdrawal (Aarabi *et al.*, 2016). The transcription factor SLIM1, a member of the EIN3-like (EIL) family, plays a central role in mediating this response. SLIM1 binds to specific promoter elements, including the UPE-box and SURE-box, to induce *LSU* gene expression under low S conditions (Maruyama-Nakashita *et al.*, 2006; Piotrowska *et al.*, 2022). In *Nicotiana tabacum*, homologous genes of the *UP9* family, particularly *UP9C*, exhibit a similar induction under S deficiency. Transcriptional activation of *UP9C* depends on promoter elements analogous to those found in Arabidopsis *LSU* genes and is regulated by NtEIL2, a SLIM1 homolog (Lewandowska *et al.*, 2010; Wawrzynska *et al.*, 2010).

Tissue-specific expression analyses using reporter constructs and RT-qPCR have shown that *LSU* transcripts accumulate primarily in young leaves and roots, particularly in the cortex and endodermis. These patterns mirror the transcriptional activity of key sulfate transporters and assimilation enzymes, suggesting a coordinated regulatory network activated under S deprivation (Ristova and Kopriva, 2022).

In monocots such as *Brachypodium distachyon*, *LSU-like* genes also display transcriptional sensitivity to S availability. *BdLSU2* is up-regulated in roots under S deficiency, whereas *BdLSU1* is down-regulated in both roots and leaves, indicating a potential functional divergence among homologs (Tombuloglu *et al.*, 2016). In broccoli (*Brassica oleracea* var. *italica*), *LSU* homologs (*BoLSU1* and *BoLSU2*) are transcriptionally induced under S deficiency. Their overexpression in Arabidopsis not only confirmed their S-responsive expression but also demonstrated their ability to modulate GSL metabolism, highlighting a broader physiological impact beyond transcriptional regulation (Yang *et al.*, 2023).

The expression of *LSU* genes under S-deficient conditions is tightly regulated by conserved *cis*-regulatory elements and transcriptional networks across plant species (Uribe *et al.*, 2022). These genes serve not only as reliable markers of S status but also as potential integrators of metabolic and signaling responses to nutrient deprivation.

### Potential roles in stress responses and signaling pathways

Beyond their well-established induction under S deficiency, LSU proteins have emerged as crucial integrators of nutrient sensing, redox regulation, and hormonal signaling in plants. Although originally classified as stress-inducible proteins of unknown function, a growing body of evidence now supports the view that LSU proteins function as molecular hubs, coordinating plant responses to a broad spectrum of environmental and physiological stressors (Fig. 2).

**Fig. 2.**
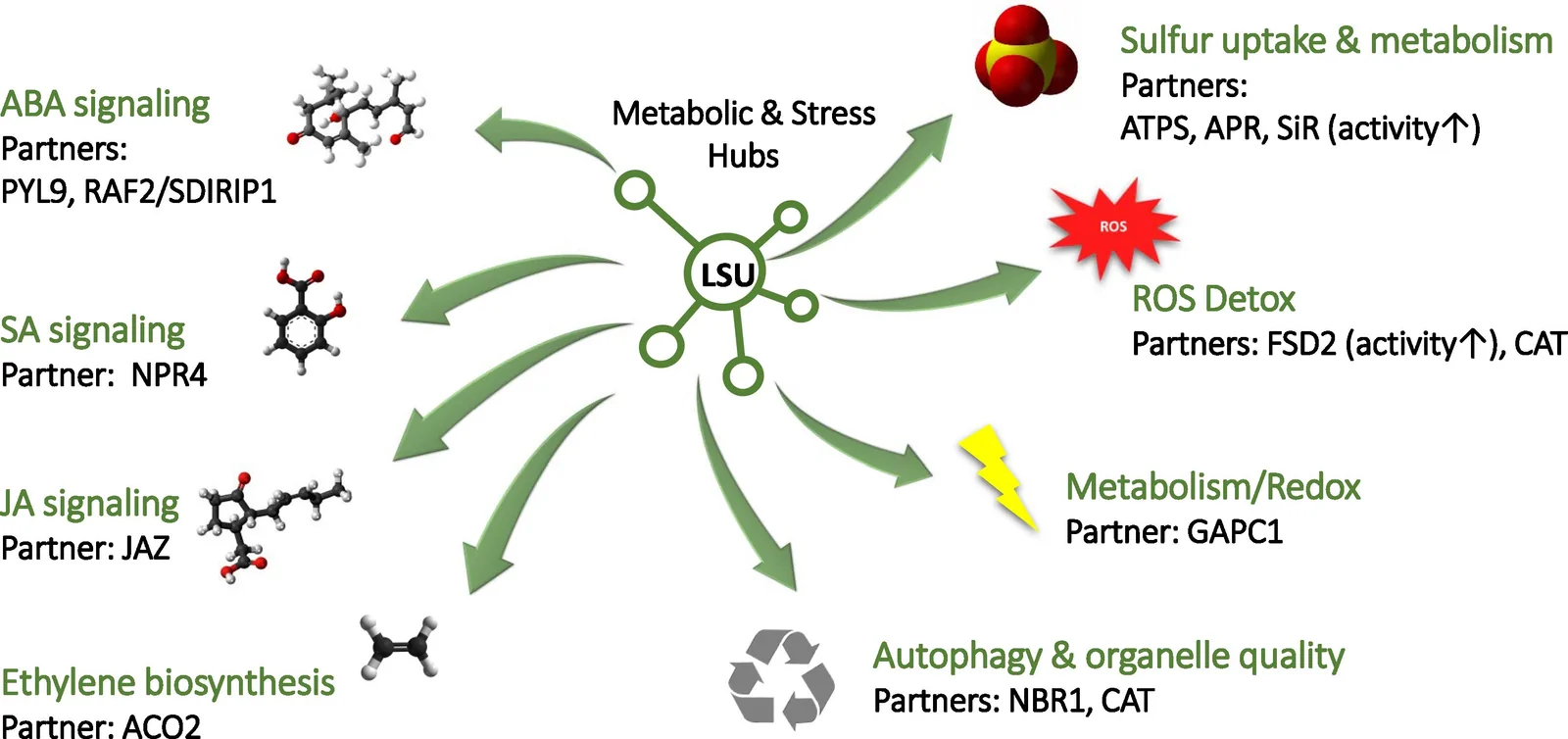
LSU proteins as regulatory hubs of stress and metabolism in plants. LSU protein partners from various metabolic pathways are shown. Abbreviations: ABA, abscisic acid; ACO2, ACC Oxidase; APR, APS Reductase; ATPS, ATP Sulfurylase; CAT, catalase; FSD2, Fe Superoxide Dismutase 2; GAPC1, Glyceraldehyde-3-Phosphate Dehydrogenase C Subunit; JA, jasmonic acid; JAZ, Jasmonate-Zim Domain; LSU, Response to Low Sulfur; NBR1, Neighbor of BRCA1 gene 1; NPR4, Nonexpresser of PR Genes 4; PYL9, Pyrabactin Resistance 1-Like 9; RAF2/SDIRIP1, Rubisco Assembly Factor 2, also known as SDIR1-Interacting Protein 1; ROS, reactive oxygen species; SA, salicylic acid; SiR, Sulfite Reductase.

Recent work pinpointed the role of LSU proteins in the S assimilation pathway (Piotrowska *et al.*, 2024b). All four Arabidopsis LSU proteins interact with multiple isoforms of enzymes involved in the sulfate-to-sulfide reduction pathway, namely ATP sulfurylases, APR, and sulfite reductase (Fig. 2). *In vitro* studies demonstrated that the interaction between LSU1 and sulfite reductase enhances enzymatic activity. Consequently, seedlings of the *lsu* quadruple mutant exhibits reduced flux through the pathway, resulting in sulfate accumulation and lower cysteine and GSH content (Piotrowska *et al.*, 2024b). Functional analyses of *lsu* mutants further highlight their systemic role in stress regulation. The *lsu* quadruple mutant in Arabidopsis shows impaired expression of genes involved not only in S assimilation—such as *SULTR1;2* and *APR3*—but also in broader stress pathways, including ROS scavenging, chaperone response, and hormone perception (Piotrowska *et al.*, 2024b). This dysregulation persists even under sufficient S conditions, underscoring LSU involvement in maintaining cellular homeostasis.

One of the most prominent roles of LSU proteins is in redox homeostasis. In Arabidopsis, LSU1 directly interacts with Fe Superoxide Dismutase 2 (FSD2), a chloroplast-localized ROS detoxifying enzyme, enhancing its enzymatic activity and contributing to oxidative stress resilience under nutrient limitation (Garcia-Molina *et al.*, 2017). Additional interactions with cytosolic Glyceraldehyde-3-Phosphate Dehydrogenase C Subunit (GAPC1), a glycolytic enzyme sensitive to oxidative modification, point to LSU involvement in integrating redox and metabolic responses during stress (Canales *et al.*, 2023).

A key discovery in *Nicotiana tabacum* revealed that the LSU-like protein UP9C physically interacts with ACC oxidase (ACO2), an enzyme catalysing the final step in ethylene biosynthesis. Plants with reduced *UP9C* expression showed impaired ethylene accumulation under S deficiency and failed to activate key ethylene-responsive transcripts (Moniuszko *et al.*, 2013). This finding indicates that LSU-like proteins contribute to fine-tuning ethylene biosynthesis and signaling under S deficiency stress. Additionally, UP9C was found to interact with the tobacco homolog of the Jasmonate-Zim-Domain (JAZ) protein family, negative regulators of jasmonic acid signaling (Sirko *et al.*, 2015). The interaction of Arabidopsis LSU proteins with components of hormonal signal transduction has been confirmed using multiple, independent protein–protein interaction approaches. Identified interaction partners include Rubisco Assembly Factor 2 (RAF2), also known as SDIR1-Interacting Protein 1 (SDIRIP1), which is implicated in ABA signaling; the ABA receptor Pyrabactin Resistance 1-Like 9 (PYL9); and the salicylic acid receptor Nonexpresser of PR Genes 4 (NPR4) (Altmann *et al.*, 2020; Niemiro *et al.*, 2020).

In addition to redox and hormonal pathways, LSU proteins intersect with general stress signaling networks (Fig. 2). Interactome analyses have identified LSU1 and LSU2 as partners of catalase 2 (CAT2), the autophagy cargo receptor NBR1, and defense-related transcription factors such as MYB51, linking LSU function with peroxisome integrity, selective autophagy, and biotic stress response (Niemiro *et al.*, 2020). A recent study by Niemiro *et al.* (2024) provided compelling evidence that LSU proteins are involved in maintaining peroxisome and catalase homeostasis. The authors demonstrated that several LSU family members physically and functionally interact with the selective autophagy cargo receptor NBR1 to regulate the abundance and turnover of catalase isoforms (particularly CAT2) in Arabidopsis. The quadruple *lsu* and the *nbr1* knockout lines exhibited abnormal accumulation of catalase and peroxisomal markers, along with hypersensitivity to oxidative stress. These results highlight a novel mechanism through which LSU proteins contribute to redox balance and organelle quality control, expanding their role in plant stress resilience beyond transcriptional regulation or partner interactions.

At the structural level, LSU proteins oligomerize via conserved coiled-coil domains into homo- and heterodimers, which likely confer interaction specificity and functional plasticity (Niemiro *et al.*, 2020). This property supports the hypothesis that LSU proteins act as scaffold-like adaptors coordinating multiple stress-related signaling pathways. Moreover, *LSU* expression and LSU interaction patterns suggest cross-talk with additional hormonal pathways, including those mediated by salicylic acid, jasmonic acid, and abscisic acid. These associations have been inferred from co-expression analyses and stress-induced transcriptomic data (Canales *et al.*, 2023).

Collectively, LSU proteins transcend their initial identification as S deficiency markers. They function as multifunctional modulators of redox balance, hormone biosynthesis, and signal transduction pathways, enabling plants to adapt to fluctuating environmental conditions (Fig. 2). Their small size, conserved structure, and versatile interaction capacity make LSU proteins promising targets for engineering enhanced stress resilience in crops.

## Future directions and conclusions

Emerging research in S metabolism and signaling is increasingly focused on integrating advanced genetic, molecular, and computational approaches to unravel complex regulatory networks. CRISPR/Cas9-mediated gene editing provides a powerful tool to dissect functional redundancy within gene families and to precisely identify key regulatory components (Piotrowska *et al.*, 2024a). Protein–protein interaction assays, ranging from the classic yeast two-hybrid system to more advanced techniques such as TurboID proximity labeling (Branon *et al.*, 2018), enable the characterization of dynamic interaction networks that underlie S sensing and signaling pathways. Single-cell transcriptomics offers unprecedented resolution to uncover tissue- or cell-type-specific expression patterns of S-related genes, advancing our understanding of spatial regulation (Ryu *et al.*, 2019). Additionally, comparative genomics across major crop species such as rice, maize, wheat, and oilseed rape can reveal conserved and divergent S-responsive genes (Fernández *et al.*, 2024), providing valuable guidance for translational research aimed at crop improvement.

In plant biology, many key components of stress and nutrient signaling networks have long remained ‘hidden players’—proteins once annotated as having unknown or hypothetical functions. Advances in genomics, proteomics, and systems biology are now uncovering that these previously uncharacterized proteins often act as key regulators of plant responses to environmental and nutritional stress. In this review we have highlighted two such ‘hidden players’ in plant S metabolism: SDI and LSU proteins. Both employ complementary strategies to help plants cope with S deficiency. SDI proteins suppress secondary S sinks, preserving the nutrient for primary metabolic processes, whereas LSU proteins play broader roles in integrating S homeostasis with stress resilience (Fig. 3).

**Fig. 3.**
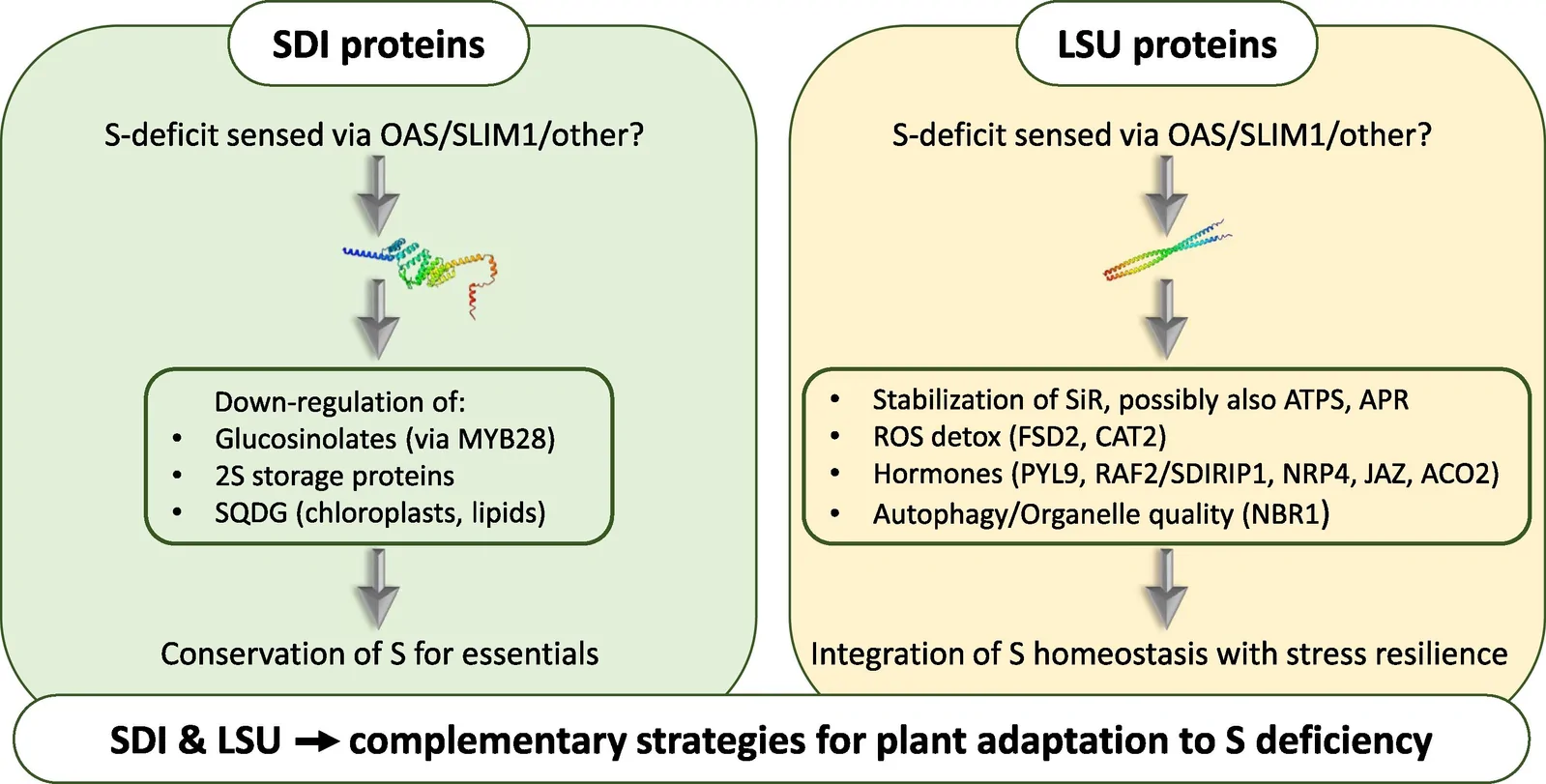
Model comparing SDI and LSU proteins function in plant response to sulfur deficit. While SDI proteins limit sulfur allocation to secondary sinks, thereby conserving it for primary metabolic activities, LSU proteins serve broader functions, coordinating sulfur homeostasis with stress tolerance mechanisms. Abbreviations: ACO2, ACC oxidase; APR, APS Reductase; ATPS, ATP Sulfurylase; CAT2, catalase 2; FSD2, Fe Superoxide Dismutase 2; JAZ, Jasmonate-Zim Domain; LSU, Response to Low Sulfur; MYB28, Myeloblastosis 28; NBR1, Neighbor of BRCA1 gene 1; NPR4, Nonexpresser of PR Genes 4; OAS, *O*-acetylserine; PYL9, Pyrabactin Resistance 1-Like 9; RAF2/SDIRIP1, Rubisco Assembly Factor 2, also known as SDIR1-Interacting Protein 1; ROS, reactive oxygen species; SDI, Sulfur Deficiency Induced; SiR, Sulfite Reductase; SLIM1, Sulfur Limitation 1; SQDG, sulfoquinovosyldiacylglycerol.

Despite recent progress, several important questions about LSU and SDI proteins still remain unanswered. For example, because LSU proteins can form oligomers, how does this affect the composition of the signaling complexes they form? Do they assemble with protein partners as dimers or as larger multimers? Do LSU proteins act as redox-sensitive scaffolds during S deficiency, modulating the activity of multiple stress-response proteins via thiol-based interactions? Additionally, how does repression by SDI proteins affect the role of LSU proteins in signal integration over the course of S starvation? Finally, what role do SDI proteins play in plant families outside the *Brassicaceae*, which are not rich in S?

Manipulating these ‘hidden players’ in S deficiency response (e.g. LSU and SDI) presents promising opportunities to enhance crop stress tolerance and yield stability under climate-related challenges such as drought, salinity, and nutrient limitation. Because these regulators affect how plants absorb and store nutrients, they could be used to biofortify food crops and improve their nutritional value. Integrating this molecular insight with advanced breeding and biotechnological approaches could pave the way for crops that are not only more resilient and sustainable but also nutritionally superior.
